# Predation Under Heat Stress: The Significance of Body Size to the Outcome of an Acarine Predator–Prey Interaction

**DOI:** 10.1002/ece3.73156

**Published:** 2026-03-31

**Authors:** Andreas Walzer, Bernhard Spangl, Lina Weissengruber, Gösta Nachman, Thomas Tscholl

**Affiliations:** ^1^ Department of Crop Sciences, Institute of Plant Protection University of Natural Resources and Life Sciences Vienna Austria; ^2^ Department of Landscape, Spatial and Infrastructure Sciences, Institute of Statistics University of Natural Resources and Life Sciences Vienna Austria; ^3^ Department of Biology University of Copenhagen Copenhagen Denmark

**Keywords:** biological control, climate change, heat sensitivity, predator–prey relationship, size at maturity, thermal mismatches

## Abstract

Due to climate warming, predator–prey interactions involving ectothermic species will more frequently be exposed to high temperatures, and asymmetric thermal shifts of the two opponents may alter the outcome of such interactions. We observed alterations of the size at maturity in the predatory mite *Phytoseiulus persimilis* and its prey, the spider mite *Tetranychus urticae*, when separately exposed to mild or extreme heat waves during juvenile development: adult predator–prey size ratios shifted from 0.83 under mild heat waves to 0.60 under extreme heat waves. Thus, firstly, experiments were conducted under optimal temperature (25°C) to evaluate the effects of shifted body size ratios on prey survival. Second, we asked whether these asymmetric thermal alterations of the adult sizes also affect prey survival under heat. Single couples of adult predator and prey females, reared under mild or extreme heat waves, were placed on a leaf disc and exposed to mild or extreme heat (corresponding to the daily temperature peaks during juvenile development) and were videotaped for 90 min. Under optimal thermal conditions, the survival rate of prey females was highest when both species were reared under extreme heat waves, emphasizing the importance of carry‐over effects from thermal juvenile environment to adult performance. At extreme heat, prey survival was 76% compared with 42% at mild heat. The predator–prey body size ratio in favor of the prey under extreme heat wave conditions was a strong predictor of predation success. This outcome is in line with the trophic sensitivity hypothesis, stating that prey is less sensitive to heat stress than its predator. We attribute this to the fact that predators become smaller and prey larger the higher the temperature is during juvenile development. Such asymmetric thermal effects may constitute a serious obstacle for achieving successful biological control of spider mites during heat waves.

## Graphical abstract

(A) The predator Phytoseiulus persimilis and its prey *Tetranychus urticae* were reared under mild or extreme heat waves from egg to adulthood, which resulted in asymmetrical thermal shifts of adult body sizes in favour of prey under extreme heat waves. (B) Single couples of adult predators and prey, reared under mild heat waves interacted at mild heat or optimum temperatures, single couples reared under extreme heat waves at extreme heat or optimum temperatures. (C) The predation risk was highest when the mites had been reared at mild heat wave conditions and tested under optimal conditions, and lowest when both the rearing and the experimental conditions were extreme, indicating strong carry‐over effects from thermal juvenile environment to adult performance.

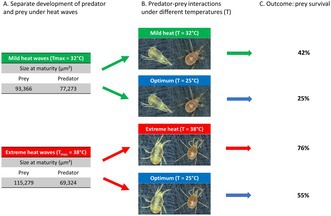

## 1 Introduction

If predators were always successful in overwhelming and killing their preferred prey after detecting them, the latter would have a permanent high risk of being eradicated by their natural enemies. In fact, predators are rarely perfect hunters as documented for several vertebrate and invertebrate predators in both terrestrial (leopards = 28% success (Bothma and Coertze 2004); lions = 29% (Van Orsdol 1984); assassin bugs = 2.5% (Bulbert et al. 2014); hornets = 69.5%–15.3% (Poidatz et al. 2023); predatory flies = 27.7% (Jaume‐Schinkel et al. 2022)) and aquatic ecosystems (gray reef sharks = 5% (Robbins and Renaud 2016); piranhas = 26% (Lowe et al. 2023); copepods = 33% (Wong 1981); dragonfly larvae = 14.7% (Bose et al. 2022)). Although there are a few examples of extraordinary high success ratios (e.g., ant‐slayer spiders = 85% (Aceves‐Aparicio et al. 2022); diving beetles = 72%–100% (Johansson and Nilsson 1992); flickers = up to 100% (Meyer 1981)), the majority of cases confirms “the life‐dinner principle” by Dawkins and Krebs (1979): the selection pressure on an attacked prey is higher than on its enemy, because the former is running for its life, the latter just for a meal. Consequently, the differential selection pressures should result in faster adaptation of traits that help the prey to escape dangers than traits enhancing the predator's hunting efficiency, thereby providing the former with an evolutionary benefit (Dawkins and Krebs 1979). Nonetheless, there are some objections against the generality of the life‐dinner principle (Humphreys and Ruxton 2020).

Over an ecological time scale, however, short‐term and unpredictable shifts of the abiotic environment can remix the cards in relation to the strength and direction of selective pressures on a predator–prey interaction. Climate change can be such a game changer, especially for ectothermic species, because the ongoing climate warming is predicted to increase the frequency, duration and intensity of heat waves (Meehl and Tebaldi 2004). Thus, periods of extremely high temperatures are likely to alter the outcome of a predator–prey interaction, because the responses to heat are frequently species‐specific (Dell et al. 2014; Tscholl, Nachman, Spangl, Serve, and Walzer 2023b; Tscholl, Nachman, Spangl, Scalmani, and Walzer 2023a). Recent studies reveal that such asymmetric thermal responses of predator and prey are often related to shifts in (i) spatial habitat use (Barton 2010; Rosenblatt et al. 2019), (ii) attack‐ and escape velocities (Kruse et al. 2008; Grigaltchik et al. 2012; Allan et al. 2015), (iii) survival rates (Pepi et al. 2018; Davidson et al. 2021), (iv) reproduction (Tscholl, Nachman, Spangl, Serve, and Walzer 2023b; Tscholl, Nachman, Spangl, Scalmani, and Walzer 2023a) and (v) developmental rates (Anderson et al. 2001; Pepi et al. 2018). In general, it seems that the prey is less heat‐sensitive than its enemy (Anderson et al. 2001; Barton 2010; Grigaltchik et al. 2012; Smolinsky and Gvozdik 2014; Allan et al. 2015; Tscholl et al. 2022, Tscholl, Nachman, Spangl, Serve, and Walzer 2023b; Tscholl, Nachman, Spangl, Scalmani, and Walzer 2023a; Walzer et al. 2022). This is in line with “the trophic sensitivity hypothesis”, stating that the lower trophic levels are less sensitive to environmental stress than the higher trophic levels (e.g., Cheng et al. 2016). On the other hand, there are also cases where the predator species profits from thermal asymmetries, resulting in higher predation rates under heat stress (Kruse et al. 2008; Pepi et al. 2018; Davidson et al. 2021).

Another aspect in relation to thermal asymmetries in developmental rates is often overlooked, namely that not only developmental times of predator and prey can be differently affected, but also their size at maturity. Such phenotypic changes may occur during a heat wave event if the involved species have very short generation times. Thus, shifts in the body size ratio in favor of the predator are likely to increase the predation risk, whereas shifts in the opposite direction may be beneficial to the prey by increasing its chances of evading attacks. However, increased body size may not necessarily be an advantage, as the relationships between temperature, body size and predation are complex. For example, larger organisms need more energy and water to survive, thereby making them more vulnerable to physiological stress. Consequently, the outcome of a predator–prey interaction depends on the balance between gains and costs of temperature‐mediated changes in body sizes (Pepi et al. 2018).

The predatory mite *Phytoseiulus persimilis* Athias‐Henriot (Acari: Phytoseiidae) is a prominent biocontrol agent, which is used worldwide in both greenhouse and field crops against the two‐spotted spider mite *Tetranychus urticae* Koch (Acari: Tetranychidae) (McMurtry and Croft 1997). The two mite species can develop from egg to adulthood within a few days ( *P. persimilis* : 4–6 days, *T. urticae* : 8–13 days), and both have a high life‐time reproductive output ( *P. persimilis* : up to 80 eggs/female, *T. urticae* : up to 160 eggs/female) (Lü et al. 2019; Tscholl et al. 2022; Tscholl, Nachman, Spangl, Serve, and Walzer 2023b; Walzer et al. 2022), resulting in high population densities within short time. However, the predator is superior with respect to the capacity for population increase (*r*_m_) thanks to its very short generation time (Sabelis 1985a). Consequently, application of *P. persimilis* usually results in an efficient and fast control of *T. urticae* (McMurtry and Croft 1997), as long as thermal conditions are optimal for the predator (daily maximum temperatures [*T*_max_] between 25°C and 32°C) (Tscholl, Nachman, Spangl, Serve, and Walzer 2023b; Tscholl, Nachman, Spangl, Scalmani, and Walzer 2023a; Walzer et al. 2015).

However, the incidence of heat waves has increased during the last decades in combination with a concurrent increase in duration and intensity, which is attributed to global climate warming (Meehl and Tebaldi 2004). These changed thermal conditions have led to more frequent spider mite outbreaks during the summer months, both in the field and in greenhouses, despite regular use of the predator *P. persimilis* . The thermal performance under constant high temperatures of predator and prey clearly favors the latter: (1) oviposition of prey peaked at 35°C, but was negligible for the predator (Urbaneja‐Bernat and Jaques 2021); (2) about 60% of prey females survived exposed to 40°C, but none of the predator females (Urbaneja‐Bernat and Jaques 2021); (3) the heat coma temperatures of adult females of predator and prey were 41.1°C and 48.7°C, respectively (Coombs and Bale 2013). Additionally, a series of lab‐experiments, conducted separately for the predator and prey under two different heat wave scenarios, defined as mild heat waves (daily maximum temperature [*T*_max_]: 32°C) and extreme heat waves (*T*_max_), provided the following main findings: (1) irrespective of species, females had a higher food intake under extreme heat waves resulting in more, but smaller eggs (Tscholl, Nachman, Spangl, Serve, and Walzer 2023b; Tscholl, Nachman, Spangl, Scalmani, and Walzer 2023a); (2) juvenile mites of both species consumed more and reached earlier adulthood under extreme heat waves, but the gain in developmental rate was higher for *T. urticae* (Tscholl et al. 2022; Walzer et al. 2022); and (3) faster development under extreme heat stress resulted in smaller body size of female predators, whereas the body size of prey females tended to become larger (Walzer et al. 2022). However, the effects of heat stress on the predation performance of *P. persimilis* , when confronted with *T. urticae* , are still unknown.

Consequently, the logical next step is to evaluate the success of the predator with respect to overwhelming and killing the prey without or with heat stress (mild or extreme). First, we started with a baseline experiment to evaluate the effects of the shifted predator–prey body size ratios (juvenile development of predator and prey to adulthood under extreme heat waves reduces their size ratios in favor of prey (Walzer et al. 2022)) on the capture success of the predator. Thus, single couples consisting of a predator and prey (adult females), which were reared under mild‐ or extreme heat waves during their juvenile development, interacted under optimal thermal conditions (constant 25°C). Second, single predator–prey couples, reared under mild‐ or extreme heat waves to adulthood, were exposed to mild heat stress (constant 32°C) or extreme heat stress (38°C), respectively. Couples exposed to mild heat were more or less similarly sized, whereas the predator was smaller than prey under extreme heat (Walzer et al. 2022). Our objectives are to verify the following hypotheses: (1) predator–prey body size ratios strongly influence the predation success under optimal temperatures leading to higher survival of the prey when the predator is small relative to prey; (2) these negative effects are boosted by extreme heat stress resulting in more inactive or unsuccessful predators, which increases the survival rates of the prey compared to mild heat stress. Alternatively, (3) the high metabolic demands of the predator (Thurling 1980) under extreme heat increase its successful attacks resulting in lower prey survival.

## 2 Materials and Methods

### 2.1 Mite Origin and Rearing

The predator *P. persimilis* and the prey *T. urticae* both originated from the company “biohelp” (Vienna, Austria), a producer and trader of natural enemies. Whole bean plants ( *Phaseolus vulgaris* L.) were used as host plants for rearing of spider mites, taking place in a climate chamber (constant 25°C ± 2°C, 60% ± 15% relative humidity [RH], 16 h:8 h [light:dark, L:D]). The rearing units of the predator consisted of acrylic plates on foam cubes placed in acrylic boxes (20 × 20 × 6 cm), half‐filled with water. Bean leaves infested with spider mites, serving as substrate and food source, were added to the plates and replaced three times per week. The boxes were placed in an incubator at constant 25°C ± 1°C, 60% ± 10% RH and 16 h:8 h (L:D).

### 2.2 Female Rearing Units, Lockable Cages and Experimental Units

We used adult females in our predator–prey interaction experiments for the following reasons: (1) adult males are smaller than females (Walzer et al. 2015, 2022; Walzer and Schausberger 2015) and therefore less voracious than females, and (2) irrespective of species, females are numerically dominant at the population level (Sabelis 1985c; Wrensch 1985). Thus, adult predator female is the most effective stage to control spider mites, and adult spider mite female is the most defensive prey stage. To produce the females for the experiments, 15–20 gravid females of each species were placed on bean leaves on foam cubes in acrylic boxes half‐filled with water (20 × 20 × 6 cm) to deposit eggs at constant 25°C. The predators were additionally provided with spider mites. After 6 h, all females were removed and the rearing units with the eggs were placed in the programmable Panasonic incubator MLR‐352H‐PE (temperature and humidity variations: ±0.5°C and ±5% RH, respectively) and exposed to simulated mild or extreme heat waves with the following temperature (*T*) and relative humidity (RH) values: (1) mild heat waves: *T*_mean_ = 22.6°C, *T*_max_ = 32.0°C, *T*_min_ = 16°C, RH_mean_ = 67.9%, RH_max_ = 85.0%, RH_min_ = 50.0%; (2) extreme heat waves: *T*_mean_ = 28.6°C; *T*_max_ = 38.0°C, *T*_min_ = 22.0°C; RH_mean_ = 60.0%, RH_max_ = 75.0%, RH_min_ = 50.0%. The photo‐period corresponded to long‐day conditions (L:D = 16 h:8 h). The climatic values on hourly basis are available in Tscholl et al. (2022). Mild and extreme heat waves should imitate heat wave conditions induced by the present and the prospective climate warming, respectively (see for details: Tscholl et al. 2022). After reaching adulthood, the females were placed singly for 24 h in lockable acrylic cages consisting of a circular cylindrical chamber (3 mm high, 15 mm diameter) (for details see Schausberger 1997; Walzer et al. 2020). This procedure allowed for selection of only fertile females, which were able to produce at least one egg during the starvation period, and to standardize the hunger level of the females used in the experiments.

**FIGURE 1:**
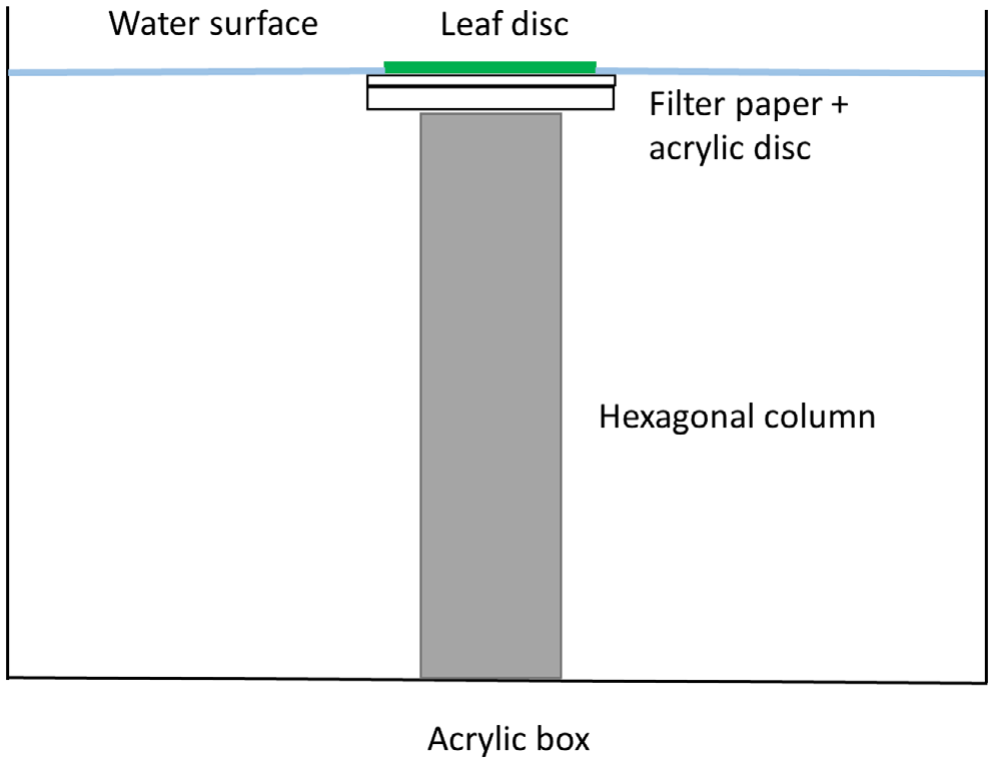
The experimental unit from the lateral view and its single components: The bean leaf disc (12 mm), the filter paper disc (14 mm), the similarly sized acrylic disc, a metallic hexagonal column (10 mm, height 30 mm) and an acrylic box (50 × 50 × 35 mm).

The experimental units consisted of an acrylic box (5 × 5 × 3.5 cm) with a hexagonal column in the middle (Figure 1). The experimental arena was placed on top of the column and consisted of an acrylic plate on which a leaf disc (12 mm) was placed with a circular piece of filter paper in between. The size of the leaf disc was large enough for the free movement of these minute small mites (0.4–0.5 mm large), but also small enough for encounters between predator and prey (Walzer and Schausberger 2013). The box was filled with water so that the periphery of the leaf disc was surrounded by water to prevent the mites from escaping (Figure 1).

### 2.3 Experiments

We carried out two series of experiments: (1) Body size effects on the predation success without heat stress, and (2) heat‐ and time effects on predator performance and prey survival. All subsequent statistical analyses were performed by means of SAS OnDemand (SAS Institute 2025). All SAS codes are available as File S1.

### 2.4 Temperature‐Mediated Effects on Body Size and Predation Efficacy

Since juvenile development under mild and extreme heat waves influences adult body size of predator and prey differently (Tscholl et al. 2022; Walzer et al. 2022), this experimental series was conducted to test whether changes in adult body size in favor of the prey affect their survival without heat stress. In the following, we used a constant temperature of 25°C as a proxy for the optimal temperature (*T*_opt_).

#### 2.4.1 Experimental Procedures

Test animals (predators: *N* = 136, prey: *N* = 140) were categorized according to how they were treated during juvenile development (Rearing Treatment [RT] = mild [predator, *N* = 66, prey, *N* = 71] or extreme [predator, *N* = 70, prey, *N* = 69]) and how the thermal conditions were during an experimental set‐up (Experimental Treatment [ET] = optimal). Adult female predators and prey couples used in the same experiment had experienced identical RT. Test animals of both species were gravid females that were starved 24 h prior to an experiment. The experiments were conducted in a programmable walk‐in climate chamber at constant 25°C (i.e., ET = optimal) and 50% RH. Four to five spider mite females were placed on the leaf disc of the experimental unit (Figure 1) 2 h prior to the start of an experiment to produce spider mite associated cues on the leaf disc. These females and their eggs were then removed and replaced by a single female prey followed by a female predator. In total, 40 pairs per RT were created and allowed to interact for 90 min, whereupon the state of the prey (*PreyState* = alive or killed) was recorded. The remaining predators (RT = mild: *N* = 26; RT = extreme: *N* = 30) and prey (RT = mild: *N* = 31; RT = extreme: *N* = 29) were killed in ethanol and their dorsal area and perimeter were measured using a microscope system (Leica DMS 1000) combined with the software Leica Application Suite X (LAS X 3.7.4.23463), both produced by Leica Microsystems, Wetzlar, Germany (Figure S1).

#### 2.4.2 Statistical Methods

- The effect of juvenile rearing conditions (RT = mild or extreme) on body size of prey and predators was analyzed by means of one‐way analysis of variance (ANOVA) using PROC GLM. Since the dorsal body area and body perimeter were found to be strongly correlated ( *P. persimilis* : *r* = 0.9913; *N* = 56; *p <* 0.0001; *T. urticae* : *r* = 0.9929; *N* = 60; *p* < 0.0001), we decided to use the dorsal body area to represent body size. To stabilize the variance, data were log‐transformed prior to the analysis. We used Q‐Q plots to check that the residuals of the transformed data were normally distributed with homoscedastic variance.
- The ratio between body size of predators and prey, reared under the same RT, was obtained as $m=\frac{\bar{y}}{\bar{x}}$, where $\bar{y}$ and $\bar{x}$ denote the average body area of *N*_*A*_ predators and *N*_*B*_ prey, respectively. As measurements of body area of predators and prey can be regarded as stochastically independent, the approximate variance of *m* was calculated as (equation 13.5.12 in Colquhoun (1971))

(1) $$V\left(m\right)\approx m^{2}\left(\frac{V\left(\bar{y}\right)}{{\bar{y}}^{2}}+\frac{V\left(\bar{x}\right)}{{\bar{x}}^{2}}\right)=m^{2}\left(\frac{V\left(y\right)/N_{A}}{{\bar{y}}^{2}}+\frac{V\left(x\right)/N_{B}}{{\bar{x}}^{2}}\right)$$

where *V*(*y*) and *V*(*x*) denote the variance of the individual measurements of body area of the *N*_*A*_ predators and *N*_*B*_ prey, respectively. 95% confidence limits for the true ratio were found as follows:

(2) $$P\left(m-t_{\nu }\sqrt{V\left(m\right)}<\mu <m+t_{\nu }\sqrt{V\left(m\right)}\right)=0.95$$

where the degrees of freedom for *t* (denoted *ν*) were found as $\nu =N_{A}+N_{B}-2$.

To test whether the difference between the ratio obtained at mild (*m*_1_) and extreme (*m*_2_) heat wave conditions could be considered as significantly different from 0, we applied a *t*‐test for two independent samples. Hence, *t* was calculated as

(3) $$t_{\nu }=\frac{m_{1}-m_{2}}{\sqrt{V\left(m_{1}\right)+V\left(m_{2}\right)}}$$

where $V\left(m_{1}\right)$ and $V\left(m_{2}\right)$ denote the variances of *m*_1_ and *m*_2_, respectively (Equation 1). The degrees of freedom for *t* were found as the sum of degrees of freedom for the two ratios (i.e., $\nu =\nu_{1}+\nu_{2}$).

iii The effect of heat wave type (RT = mild or extreme) during juvenile development on the number of adult prey being alive or dead at the end of the interaction period (90 min) was analyzed by means of a *χ* ^2^‐test for two independent samples using PROC FREQ.

### 2.5 Predation Under Heat Stress

These experiments were carried out to examine heat effects on the predation efficacy of the predator and the resulting survival probability of the prey.

#### 2.5.1 Experimental Procedures

The RT conditions of the females (i.e., mild or extreme heat waves), the state of the females (gravid, starved), the experimental units, the experimental location (i.e., a walk‐in climate chamber) and the experimental duration (90 min) were identical to the former experiments. The differences from the former experiments were: (1) the ET conditions of the interacting females were either mild (constant 32°C) or extreme heat stress (38°C), corresponding to the daily *T*_max_ values experienced during their juvenile development (RT) (Figure S2), (2) the behavior of the females was videotaped (mild heat stress: *N* = 36, extreme heat stress: *N* = 38) by means of an integrated digital camera (DMS 1000, Leica GmbH, Germany), (3) we also made recordings of predators and prey occurring without an opponent (mild heat stress, prey: *N* = 23, predator: *N* = 29, extreme heat stress, prey: *N* = 24, predator: *N* = 28) (Figure S2) These recordings served as controls, (4) all variables (described in detail in the statistical methods) were obtained directly from the video recordings.

#### 2.5.2 Statistical Methods

- *χ* ^2^‐tests for two independent samples (PROC FREQ) were used to analyze whether heat treatment (ET = mild, extreme) affected the final state of the female prey (killed or alive), measured by the dependent variable *PreyState*.
- The performance of female predators, expressed by the variable *PredatorState*, after exposure to either mild or extreme heat was compared by means of a *G*‐test (PROC FREQ). The state of an individual predator at the end of a recording was categorized as either *Inactive* (no attacks), *Loser* (all attacks were unsuccessful), or *Winner* (at least one successful attack). An encounter between the predator and prey was considered as an attack if the predator was touching the prey with the pedipalps or first pair of legs (Walzer, Paulus, and Schausberger 2004). The *G‐*test was chosen because some of the expected frequencies were < 5.
- The effect of heat on the predator's attack rate was modeled by means of a generalized linear model (PROC GENMOD). Since the total number of attacks per predator (*Attacks*) is a non‐negative integer variable, we assumed that the underlying distribution was either random (if average ≈ variance) or aggregated (if average < variance). The former was modeled by setting *dist* = Poisson and the latter by setting *dist* = negbin. In both cases, the link function was set to be logarithmic and the scaling factor set to *dscale* to account for over‐ or under‐dispersion. The success ratio of predators exposed to mild or extreme heat was expressed as the total number of killed prey divided by the total number of attacks.
- The Kaplan–Meier survival analysis (PROC LIFETEST) was used to assess the probability that a prey survives until a given time (defined by *SurvTime*). The entire experimental period (5400 s) was divided into 10 time intervals of 9 min, and the number of prey killed in each interval was calculated. Individuals being alive at the end of an experiment (i.e., *SurvTime* > 90 min) were censored from the analysis. Data were stratified according to heat stress (ET = mild or extreme). The difference between the two strata with respect to attack risk was tested by means of log‐rank test, Wilcoxon's two‐sample test and log‐likelihood test. Epanechnikov kernel‐smoothed hazard functions were used to model the relationship between time and attack risk for each temperature separately.
- Logistic regression was used to test the relative influences of RT and ET on survival probability of prey during the entire interaction period (90 min). Empirical survival rates were available for four out of six possible treatment combinations, namely (1) RT = mild, ET = mild; (2) RT = extreme, ET = extreme; (3) RT = mild, ET = optimal; and (4) RT = extreme, ET = optimal. The logistic model fitted to data was.

(4) $$S/N=\frac{e^{\beta_{0}+\beta_{1}x_{1}+\beta_{2}x_{2}+\beta_{3}x_{3}}}{1+e^{\beta_{0}+\beta_{1}x_{1}+\beta_{2}x_{2}+\beta_{3}x_{3}}}$$

where *N* is the number of prey exposed to a given combination of RT and ET and *S* the number surviving the experiment. *x*_1_, *x*_2_ and *x*_3_ are dummy variables used to represent the various treatment combinations (i.e., *x*_1_ = 1 if RT = extreme or otherwise 0; *x*_2_ = 1 if ET = extreme or otherwise 0; *x*_3_ = 1 if ET = mild or otherwise 0). $\beta_{0}$, $\beta_{1}$, $\beta_{2}$ and $\beta_{3}$ are the model's parameters, where $\beta_{0}$ expresses the expected survival probability when *x*_1_ = *x*_2_ = *x*_3_ = 0 (i.e., when RT = mild and ET = optimal), $\beta_{1}$ expresses the effect of RT = extreme relative to RT = mild, $\beta_{2}$ expresses the effect of ET = extreme relative to ET = optimal and $\beta_{3}$ expresses the effect of ET = mild relative to ET = optimal.

The underlying distribution was assumed to be binomial (i.e., *dist* = Bin) with *link* = logit. Finally, to adjust for potential over‐ or under‐dispersion, the scaling factor was set to *dscale*. Since the experiments were not carried out as a full factorial design, the model could not include an interaction term between RT and ET. However, the model enabled us to predict the expected survival rate of prey exposed to RT = mild and ET = extreme, and to RT = extreme and ET = mild, provided the combined effect of the two treatments is assumed to be additive.

## 3 Results

### 3.1 Temperature‐Mediated Effects on Body Size and Predation Efficacy

**FIGURE 2:**
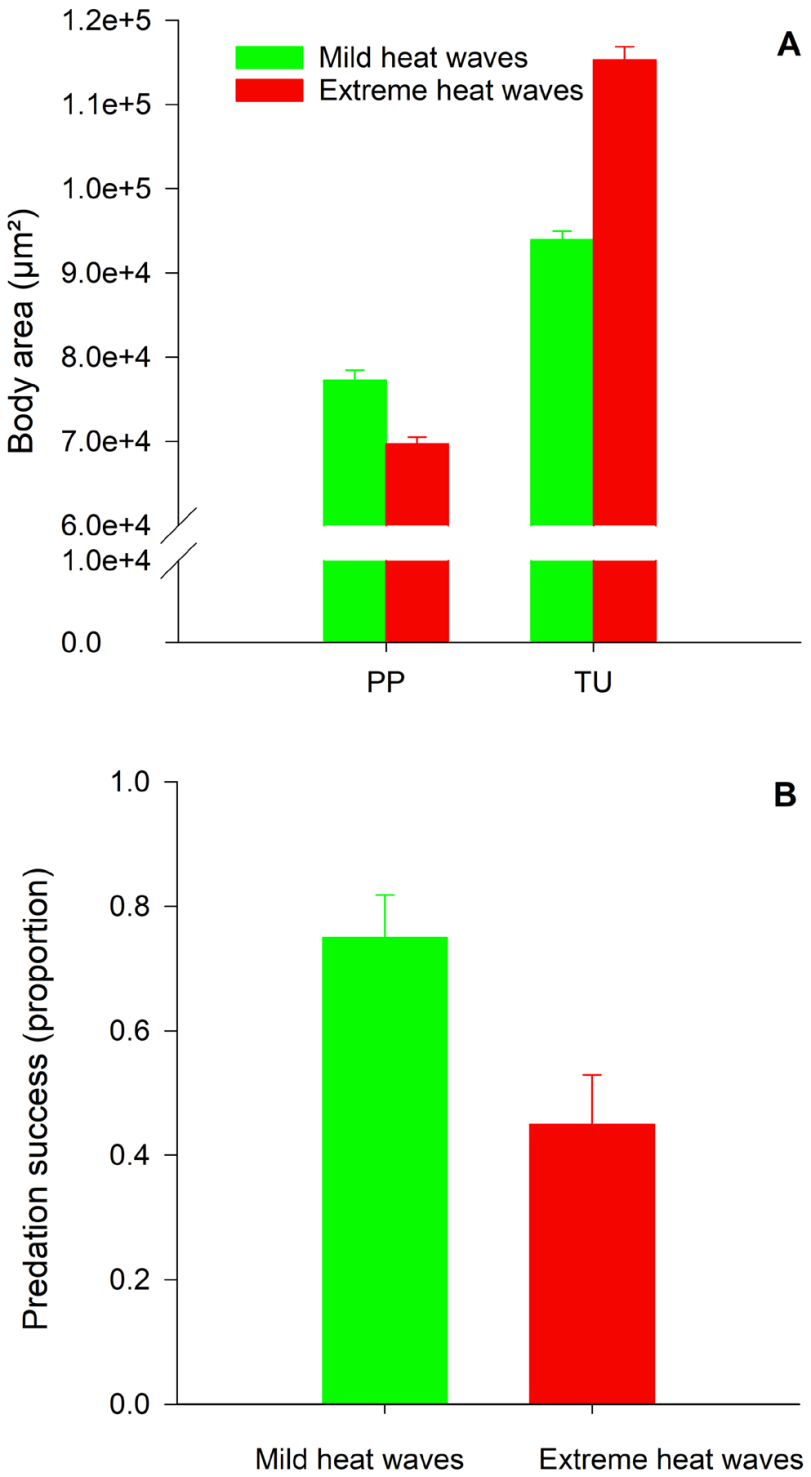
(A) Body area (with 95% confidence limits) of the female predator *P. persimilis* (PP) and its prey *T. urticae* (TU) reared under mild or extreme heat wave conditions. (B) Predation success of female *P. persimilis* on female *T. urticae* at 25°C during 90 min. Both species were reared under the same heat wave conditions (mild or extreme). Vertical lines on top of the bars show the standard error of the proportion of killed prey.

- Irrespective of species, none of the females deposited an egg during the experimental period. All predator–prey couples survived until the end of the experiments, when the predator was unable to overwhelm the prey. The GLM showed that the juvenile rearing conditions (RT) explained 36.49% of the total variation in log (body area) for the adult female predators (*F*_1,54_ = 31.04; *p* < 0.0001) and 70.86% for the adult female prey (*F*_1,58_ = 141.0; *p* < 0.0001). The results clearly demonstrate that the thermal conditions during juvenile development influence the adult body size of predators and prey differently: thus, exposure to extreme heat waves during juvenile development decreased the body area of the predators by 10.3%, but increased the body area of the prey by 23.5% compared with the body sizes of mites exposed to mild heat waves (Figure 2A).
- The ratio between the average body area of predator and prey females reared under mild heat wave conditions was 77,273 μm^2^/93,366 μm^2^ = 0.828 (95% CL: 0.796–0.859), but only 69,324 μm^2^/115,279 μm^2^ = 0.601 (95% CL: 0.579–0.623) when the two species were reared under extreme heat wave conditions. It corresponds to a decrease of 27.4%, which is highly significant (*t*_112_ = 11.73; *p <* 0.0001).
- Predator females reared under mild conditions, and therefore larger than the females reared under extreme conditions, launched significantly more successful attacks compared with the performance of the smaller females when both groups were tested at optimal thermal conditions (25°C) ($\chi_{1}^{2}$ = 7.50; *p* = 0.0062). Thus, the proportion of surviving prey when the predators were large, was only 25% (10 out of 40) compared with 55% (22 out of 40 prey) when the predators were smaller (Figure 2B).

### 3.2 Predation Under Heat Stress

**FIGURE 3:**
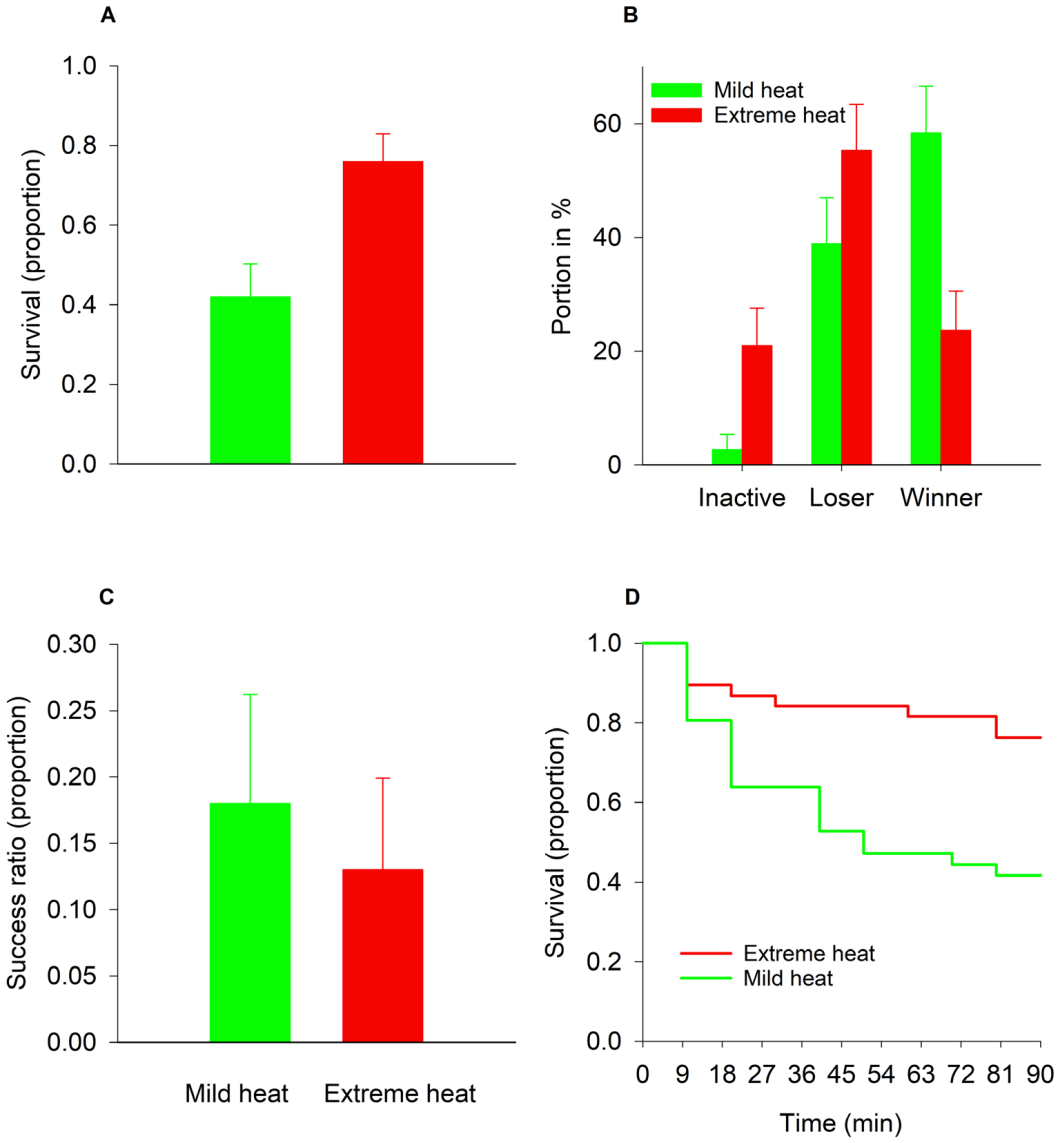
Effects of heat on (A) the survival probability of the prey *T. urticae* , (B) the behavioral pattern of the predator *P. persimilis* (inactive: No attacks, loser: Only non‐successful attacks, winner: At least one successful attack), (C) predation success (number of successful attacks/number of attacks), and (D) time‐dependent effect of heat on the survival chance of a female prey confronted by a female predator over 90 min on a bean leaf disc. Vertical lines on top of the bars show the standard error of the observed proportions.

- Irrespective of species and temperature, none of the females laid an egg during the experimental period. Furthermore, all individuals survived when they were not confronted with their opponent species (32°C, prey: *N* = 23, predator: *N* = 29; 38°C, prey: *N* = 24, predator: *N* = 28). When a prey was together with a predator, its survival probability was strongly affected by the temperature (${Χ}_{1}^{2}$ = 9.207, *p* = 0.0024). Thus, only 42% (15 out of 36) prey females survived at 32°C, whereas 76% (29 out of 38) of the females were still alive after 90 min when the temperature was 38°C (Figure 3A). All interacting predator females survived, irrespective of heat stress (32°C: *N* = 36, 38°C: *N* = 38).
- The behavior of *P. persimilis* females was significantly influenced by heat stress (${Χ}_{2}^{2}$ = 12.40, *p* = 0.0019). When exposed to 38°C, 21% of the predators (8 out of 38 individuals) were inactive, while 55% (21/38) were loser (failed to overwhelm a prey female despite attacks) and only 24% (9/38) were winner (able to kill a prey female). In contrast, the corresponding values at 32°C were 3% (1/36) inactive, 39% (14/36) loser and 58% (21/36) winner (Figure 3B).

Independently of heat stress, all successful attacks were characterized by the prey losing either partial or complete leaf contact during an attack so that the predator sucked the prey out from their lateral or ventral body regions. Predator attacks from the dorsal side of the prey were never successful.

iii The average number of attacks per predator female was 3.139 (SE = 0.521; *N* = 36) at 32°C and 1.789 (SE = 0.331; *N* = 38) at 38°C. Since the variances (9.780 at 32°C and 4.171 at 38°C) were two to three times higher than the averages, it indicated that the negative binomial distribution (NBD) was more suitable than the Poisson distribution to describe the uneven pattern of attacks, that is, a few predators accounted for the majority of attacks. When the distribution of attacks per predator was modeled by means of a NBD, the scaling factor was found to be 0.4953, which indicated a moderate under‐dispersion relative to the NBD. The effect of heat stress on attacks per female predator was significant (PROC GENMOD: $\chi_{1}^{2}$ = 5.90; *p* = 0.0152) indicating a higher success ratio of the predators under mild heat stress (Figure 3C).
iv The difference between the two survival curves was significant (log‐rank test: $\chi_{1}^{2}$ = 9.270; *p* = 0.0023; Wilcoxson: $\chi_{1}^{2}$ = 8.346; *p* = 0.0039; log‐likelihood test: $\chi_{1}^{2}$ = 10.944; *p* = 0.0009), which means that the prey's chance of survival was much higher at 38°C than at 32°C (Figure 3D). The Epanechnikov hazard functions were found to decline with time (especially at 38°C), indicating that the ability of a predator to carry out a successful attack decreased with time independently of temperature. Moreover, the hazard function for 38°C approached 0, meaning that the predators suffered so severely from heat stress that they became unable to launch a successful attack after 90 min exposure. Contrary, the predators were still able to perform reasonably well under mild heat stress.
v The logistic model shows that the juvenile rearing temperature (RT) had a strong effect on prey survival probability ($\chi_{1}^{2}$ = 7.64; *p* = 0.0057) compared with the weaker effect of the experimental temperature (ET) ($\chi_{2}^{2}$ = 6.36; *p* = 0.0415). The estimated values of the model's parameters are shown in Table 1. The fact that $\beta_{1}$ was found to be positive and significantly different from 0 indicates that extreme heat wave conditions during rearing had a strong negative impact on the predation efficiency compared with mild heat wave conditions. $\beta_{2}$ was found to be positive and marginally significant, indicating the survival probability of the prey was slightly higher when experimental conditions were extreme (38°C) compared with experiments conducted at 25°C. $\beta_{3}$ was also positive but not significantly different from 0, which indicates that survival probability was more or less the same at 32°C and 25°C. Predation risk was highest when the mites had been reared at mild heat wave conditions and tested under optimal conditions, and lowest when both the rearing and the experimental conditions were extreme (Table 2).

**TABLE 1:** Estimated values and standard errors (SE) of the parameters in Equation (4) associated with the three dummy variables *x*_1_, *x*_2_ and *x*_3_ used to model the six possible combinations of rearing temperature (i.e., RT = “mild” and “extreme”) and experimental temperature (i.e., ET = “optimal”, “mild” and “extreme”). *P* is the probability of obtaining a parameter value that is numerically equal to or larger than the observed one by chance assuming that the true value is 0.

Parameter	Estimate	SE	Wald's $\chi_{1}^{2}$	*p*
*β*_0_	−1.0986	0.3651	9.05	0.0026
*β*_1_	1.2993	0.4841	7.20	0.0073
*β*_2_	0.9694	0.4966	3.81	0.0509
*β*_3_	0.7621	0.4976	2.35	0.1256

**TABLE 2:** Observed and predicted survival probabilities for female *T. urticae* exposed to predation by female *P. persimilis* .

RT	ET	Obs. survival probability	Pred. survival probability	Lower 95% CL	Upper 95% CL
Mild	Optimal	0.250	0.250^a^	0.140	0.405
Mild	Mild	0.417	0.417^ab^	0.269	0.581
Mild	Extreme	—	0.468^abc^	0.208	0.746
Extreme	Optimal	0.550	0.550^abc^	0.396	0.695
Extreme	Mild	—	0.724^bc^	0.452	0.893
Extreme	Extreme	0.763	0.763^c^	0.604	0.872

*Note:* Values are arranged in increasing order with respect to prey survival probability (reflecting declining predator efficacy). Combinations of treatments that were not tested experimentally are indicated by a “—”. The predicted survival probabilities were obtained from the model assuming that the combined effect of RT and ET on survival is additive. Predicted survival probabilities followed by the same letter are not significantly different (*p >* 0.05).

Abbreviations: ET, experimental temperature; RT, rearing temperature.

## 4 Discussion

Our study reveals that extreme heat stress (38°C) has a strong negative impact on the efficacy of the predatory mite *P. persimilis* feeding on its preferred prey, the two‐spotted spider mite *T. urticae* . The predators attacked prey less often and were either more inactive or less successful at extreme heat compared with their performance at mild heat (32°C). The prey's instantaneous risk of being killed decreased steeply over time and was virtually zero after 90 min exposure to extreme heat, in contrast to when the prey was pursued by predators under mild heat stress. Consequently, the majority of prey (76%) survived encounters with the predator under extreme heat stress compared with only 42% at mild heat. In concert with other ectothermic predator–prey interactions under heat stress (ground beetles—fruit flies (Kruse et al. 2008), spiders—grasshoppers (Barton 2011); Australian bass—eastern mosquitofish (Grigaltchik et al. 2012)), our outcome agrees with the predictions of the trophic sensitivity hypothesis, stating that a prey is less sensitive to heat stress than its predator (Cheng et al. 2016).

Similar to the findings of Tscholl, Nachman, Spangl, Serve, and Walzer (2023b) and Tscholl, Nachman, Spangl, Scalmani, and Walzer (2023a), we found that female predators experiencing extreme heat wave conditions during juvenile development were 10.3% smaller at adulthood compared with individuals developing under mild heat wave conditions. Contrary, the sizes of female prey increased by 23.5% under the same conditions. Consequently, the size ratio between adult female predators and prey declined by 27.4% (from 0.828 to 0.601) when the two species had developed under extreme heat wave conditions compared with mild heat waves. When predators and prey both developed under mild heat wave conditions and were later confronted with each other as adults at optimal conditions (25°C), we found that 75% of the prey were killed within 90 min in contrast to only 45% prey mortality when the mites had been reared under extreme heat wave conditions. Since the two groups of mites were tested under optimal conditions, it demonstrates that temperature conditions during the juvenile stages have a significant effect on the predation efficacy of adult predators. We attribute this to the fact that the predators become smaller, and the prey larger, the higher the temperature is during juvenile development, which will benefit the prey when it is attacked by a predator. Though we cannot exclude that other carry‐over effects from the juvenile stage affect the performance as adults, body size ratio is nevertheless the most conspicuous factor and the best predictor of whether a prey will survive a confrontation with a predator.

### 4.1 The Significance of the Predator–Prey Body Size Ratios

Single, actively hunting predators without the ability of using venoms are often larger than their preferred prey (Sabelis 1992). In our study, the predator had a high attack success under mild heat conditions, despite the female prey was slightly larger than the predator. Other cases of arthropod predators killing larger prey are also documented (hemipteran bugs (Nakazawa et al. 2013); lacewing larvae (Samková et al. 2023); firefly larvae (Krämer et al. 2024)) with some highly spectacular predator–prey interactions (e.g., snakes and scorpions were overwhelmed and consumed by smaller spiders (Nyffeler and Gibbons 2021) and assassin bugs (Lira et al. 2016), respectively), but these predators have one trait in common: extra‐oral digestion (Cohen 1995). This type of digestion is widespread in arthropod predators, where digestive enzymes induced by the predator change the body content to a fluid state allowing for faster intake of high‐quality food compared to intra‐oral digestion. The second benefit is that the predator can also overwhelm larger prey because of the transferred paralyzing substances, which immobilizes the prey (Cohen 1995) and minimizes the risk of injuries from struggling prey (Shatrov 2005). Extra‐oral digestion was proved for the predatory soil mite *Pergamasus longicornis* (Acari: Parasitidae) (Bowman 2014), and there is also some evidence that *Phytoseiulus fragariae*, a close relative to *P. persimilis* , uses this digestion method as well (Flechtmann and McMurtry 1992). It therefore seems likely that the ability of *P. persimilis* to overwhelm and kill larger prey than itself relies on extra‐oral digestion.

However, the attack success of *P. persimilis* females decreased as the body size differences became still larger in favor of the prey under extreme heat. We observed that successful *P. persimilis* females were able to pull down the prey so that the predator penetrated them from the lateral or ventral body regions, whereas attacks from the dorsal side of the prey were unsuccessful. It seems likely that these observations are directly and indirectly connected with the body sizes of both predator and prey. First, significantly larger prey can perform stronger physical resistance against the smaller predator, which limits the ability of the predator to floor the prey. Second, larger prey body size may also mean longer dorsal setae of the prey, which can serve as an effective mechanical defense mechanism against predators, hindering them from penetrating the cuticula on the dorsal body regions of the prey as documented in the spider mite *Panonychus ulmi* (Yano and Shirotsuka 2013).

In our experiments, the predator suffered not only from smaller body size but was concurrently exposed to extreme heat stress. Whether the thermal asymmetries in body sizes of predator and prey are correlated with other trait shifts (velocity, activity) in favor of prey under extreme heat stress will be addressed in a following paper.

### 4.2 Potential Implications at Population Level

Theoretically, the ability of *P. persimilis* to control spider mite populations is expected to decrease under extreme heat, since the majority of prey females succeed to withstand predator attacks and at the same time continue to lay eggs. Under natural conditions, however, the predators have various opportunities to optimize energy intake for their own survival and prospective reproduction (Stephens and Krebs 1986). For instance, prey patches of spider mites are usually composed of eggs, juveniles and adults, allowing the predator to select the most profitable prey item in terms of net energy gain. In general, *P. persimilis* prefers to feed on spider mite eggs in choice‐experiments (Blackwood et al. 2001; Moghadasi et al. 2013). This is likely due to the handling times of the predator, which are about ten times shorter for spider mite eggs compared with adult prey females (Sabelis 1981). On the other hand, the number of consumed prey items required to produce a single predator egg is much higher when a predator feeds on spider mite eggs compared with adult prey (8.5 eggs versus 1.5 females (Vanas et al. 2006; Xue et al. 2009)). Interestingly, the success ratio of adult *P. persimilis* females attacking adult female spider mites has been found to be higher when the prey was located in the complex and three‐dimensional web on the underside of leaves and not directly on the leaf surface (Sabelis 1981). As a specialist predator of spider mites, *P. persimilis* can easily move around on the silk threads of a web, thereby guiding the predator to profitable prey patches (Sabelis 1985b). Since the spider mite females are in the web without contact to the leaf surface, they are more easily overwhelmed by the predator because of their unprotected ventral body regions. Furthermore, prey preferences may change in accordance with a predator's nutritional and/or reproductive state and so may the handling time of individual prey items. Many predators are able to adjust their choice of prey and the handling time per prey item in accordance with their current nutritional and reproductive state as demonstrated in some ectothermic species (e.g., ground beetles, spiders (Mayntz et al. 2005)). Young *P. persimilis* predominantly attack immature stages of spider mites, whereas adult *P. persimilis* may also include adult prey in their diet (Moghadasi et al. 2013). By eliminating the reproductive units of the prey species (i.e., the adult females), *P. persimilis* has the potential to be a highly efficient biocontrol agent, at least as long as the ambient temperature and humidity conditions are tolerable for its activity.

When the ambient temperature rises, individual predators will not only become smaller, but at the same time they will be confronted with larger prey. This by itself will impede attack efficiency against adult prey and instead make the younger prey stages, and especially the eggs, more attractive as a food source. However, high temperatures may also directly affect the performance of *P. persimilis* , causing heat stress, paralysis and eventual death (Coombs and Bale, 2013). Such physiological phenomena will also affect *T. urticae* , but typically at higher temperatures (Coombs and Bale, 2013). To survive during periods of harmful temperatures, ectothermic predators may choose between two strategies: (i) they may either stay put and reduce activity to a minimum in order to save energy and avoid desiccation or (ii) they may try to escape to thermal refuges with more benign climatic conditions, but often with an inadequate supply of prey (e.g., Martín and López 2010; Ortega et al. 2023). Evidence obtained from small‐scale laboratory experiments (experimental units: single bean leaves) indicates that *P. persimilis* females apply the second strategy as 74%–77% of the predator individuals left spider mite prey patches under extreme heat waves in spite of ample food supply (Tscholl, Nachman, Spangl, Serve, and Walzer 2023b; Tscholl, Nachman, Spangl, Scalmani, and Walzer 2023a). At the crop level, the microclimate of the preferred habitat for the predator and prey, the underside of leaves, can also affect their interaction under heat waves (Caillon et al. 2014; Ma et al. 2021). The leaf temperatures are dependent on multiple factors such as the surface, size, shape and position of the leaves. For example, the leaf temperature is 8°C higher and 1°C lower than the ambient temperatures in sunny and shaded apple leaves, respectively (Saudreau et al. 2017). Consequently, predatory mites may temporarily escape lethal temperatures by moving to shaded areas inside the canopy, leading to diurnal and/or seasonal variation in the spatial distribution of the predators (Villanueva and Childers 2005; Pérez‐Sayas et al. 2018). Such temperature‐mediated movement patterns may result in spatial separation between predators and prey as documented for the spider mite *Eutetranychus orientalis* (Acari: Tetranychidae) and its predator *Euseius stipulatus* (Acari: Phytoseiidae) in citrus orchards. The prey preferred the outer and upper leaves, the predator inner and lower leaves (Vela et al. 2017). Whether such a distribution pattern is also exhibited by the predator *P. persimilis* and its prey *T. urticae* under heat waves, probably leading to an inefficient spider mite control, remains an open question.

## Author Contributions

**Andreas Walzer:** conceptualization (lead), data curation (supporting), funding acquisition (lead), investigation (lead), methodology (lead), project administration (lead), supervision (lead), visualization (lead), writing – original draft (lead), writing – review and editing (equal). **Bernhard Spangl:** formal analysis (lead), writing – review and editing (equal). **Lina Weissengruber:** data curation (lead), investigation (supporting), writing – review and editing (equal). **Gösta Nachman:** formal analysis (lead), writing – review and editing (equal). **Thomas Tscholl:** data curation (supporting), investigation (supporting), writing – review and editing (equal).

## Funding

This work was supported by the Austrian Science Fund, P32474.

## Conflicts of Interest

The authors declare no conflicts of interest.

## Supporting information


**Figure S1:** Experimental set‐up of experiment 1: Temperature‐mediated effects on body size and predation efficacy.
**Figure S2:** Experimental set‐up of experiment 2: Predation under heat stress.
**Data S1:** ece373156‐sup‐0001‐supinfo.zip.

## Data Availability

The data that support the findings of this study are openly available in Zenodo at https://doi.org/10.5281/zenodo.15709870 (accessed on 21.06.2025).
