# Supplementary material for: Predation Under Heat Stress: The Significance of Body Size to the Outcome of an Acarine Predator–Prey Interaction
Source: Ecol Evol. 2026 Mar 31;16(4):e73156. doi: 10.1002/ece3.73156 (PMC13106986; doi:10.1002/ece3.73156)
Supplement: Supplementary file 1 — Figure S1: Experimental set‐up of experiment 1: Temperature‐mediated effects on body size and predation efficacy. Figure S2: Experimental set‐up of experiment 2: Predation under heat stress. Data S1: ece373156‐sup‐0001‐supinfo.zip. [file ECE3-16-e73156-s001.zip › ece373156-sup-0003-File S1.pdf]

SAS codes

```
/* Analyses of temperature-mediated effects on body size */
/* Program name: "BodySizes.sas" */

%web_drop_table(WORK.IMPORT);

/* Import data from an Excel file */
FILENAME REFFILE '/home/u44778551/Tscholl and Walzer/Behavioural experiments/Body
size/BodySizeMeasurements.xlsx';

PROC IMPORT DATAFILE=REFFILE
        DBMS=XLSX
        OUT=WORK.IMPORT;
        GETNAMES=YES;
        SHEET = "SAS data";
RUN;

PROC CONTENTS DATA=WORK.IMPORT; RUN;

%web_open_table(WORK.IMPORT);

/* Make a new data set called "MyData" and add log(BodyArea) */
data MyData;
set work.import;

logArea = log10(BodyArea);

/* Summary statistics */
proc sort data = MyData;
by Species HeatWave;
run;

/* Find averages and variances of area and periphery of mite bodies for each species and
HeatWave type */
proc means data = MyData mean var std n;
var BodyArea Perimeter;
output out = Summary mean = AvgBodyArea AvgPerimeter var = VarBodyArea varPerimenter std
= stdBodyArea stdPerimeter n = ObsBodyArea obsPerimeter;
by Species HeatWave;
run;

/* Calculate confidence limits for ratios of BodyAreas for each Species and HeatWave */
proc means data = MyData mean var n;
var BodyArea;
output out = Summary mean = AvgBodyArea var = VarBodyArea n = Obs;
by Species Heatwave;
run;

/* export data as an Excel file in order to calculate confidence limits for ratios*/
PROC EXPORT
```

```
 data = Summary
 dbms =xlsx
 outfile = '/home/u44778551/Tscholl and Walzer/Behavioural experiments/Body
size/Satherwaith data'
 REPLACE;
RUN;

/* correlation between body area and perimeter */
proc corr data = MyData;
var BodyArea Perimeter;
by Species;
run;

/* Effect of temperature on bodysize */
proc glm data = MyData;
Class Heatwave;
model logArea = Heatwave /solution;
by Species;
run;
```

```
/* Analyses of predation experiments */
/* Program name: "Attack success.sas " */

/* Import data from an Excel file */
%web_drop_table(WORK.IMPORT);
FILENAME REFFILE '/home/u44778551/Tscholl and Walzer/Behavioural
experiments/Predation/Predation experiments.xlsx';
PROC IMPORT DATAFILE=REFFILE
	DBMS=XLSX
	OUT=WORK.IMPORT;
	GETNAMES=YES;
	SHEET = "SAS data";
RUN;
PROC CONTENTS DATA=WORK.IMPORT; RUN;
%web_open_table(WORK.IMPORT);

proc print data = work.import;
run;

/* Make a new data set called "MyData" */
data MyData;
set work.import;

if Attacks = 0 then Performance = 'Inactive';
if (Attacks > 0) and (Survival = 'Alive') then Performance = 'Loser';
if (Attacks > 0) and (Survival = 'Killed') then Performance = 'Winner';
if Temperature = 32 then HeatWave = 'Mild'; else HeatWave = 'Extreme';

/* Effect of temperature on attack rate */
proc freq data = MyData;
tables HeatWave*Survival / chisq;
run;

/* Effect of temperature on predator performance */
proc freq data = MyData;
tables HeatWave*Performance /chisq expected;
run;

/* effect of temperature on the number of attacks per predator */
proc sort data = MyData;
by HeatWave;
run;

/* Average number of attacks per predator */
proc means data = Mydata mean sum var stderr n;
var Attacks;
output out = summary mean = avgAttack sum = sumAttacks var = varAttacks stderr = seAttacks n =
obs;
by HeatWave;
run;
```

```
/* Analysis of prey survival when exposed to a predator */
/* Program name "Survival.sas" */

%web_drop_table(WORK.IMPORT);

FILENAME REFFILE '/home/u44778551/Tscholl and Walzer/Behavioural
experiments/Survival/Survival data.xlsx';

PROC IMPORT DATAFILE=REFFILE
        DBMS=XLSX
        OUT=WORK.IMPORT;
        GETNAMES=YES;
        SHEET = "Frequencies";
RUN;

PROC CONTENTS DATA=WORK.IMPORT; RUN;

%web_open_table(WORK.IMPORT);

data survival;
set work.import;

Counts = Survived+Died;  /* Total number of events */

/* Test whether predator efficacy is affected by rearing (RT)and experimental (ET) temperature */
/*
Treatment        RT        ET
1               Mild       Opt
2               Extreme    Opt
3               Mild       Mild
4               Extreme    Extreme
5               Mild       Extreme
6               Extreme    Mild
*/


proc genmod data = survival descending;
class Treatment;
model Survived/counts = Treatment /dist = bin link= logit dscale type3;
lsmeans Treatment / adjust = tukey lines ;
contrast '1 against 2' Treatment 1 -1 0 0;
contrast '1 against 3' Treatment 1 0 -1 0;
contrast '2 against 4' Treatment 0 1 0 -1;
contrast '5 against 6' Treatment 0 0 1 -1;
contrast '1 and 3 against 2 and 4' Treatment 1 -1 1 -1;
contrast '1 and 2 against 3' Treatment 1 1 -2 0;
contrast '1 and 2 against 4' Treatment 1 1 0 -2;
run;

/* Test whether prey survival is mostly determined by RT or ET */
proc genmod data = survival;
```
