# Supplementary figures and images for: Predation Under Heat Stress: The Significance of Body Size to the Outcome of an Acarine Predator–Prey Interaction
Source: Ecol Evol. 2026 Mar 31;16(4):e73156. doi: 10.1002/ece3.73156 (PMC13106986; doi:10.1002/ece3.73156)

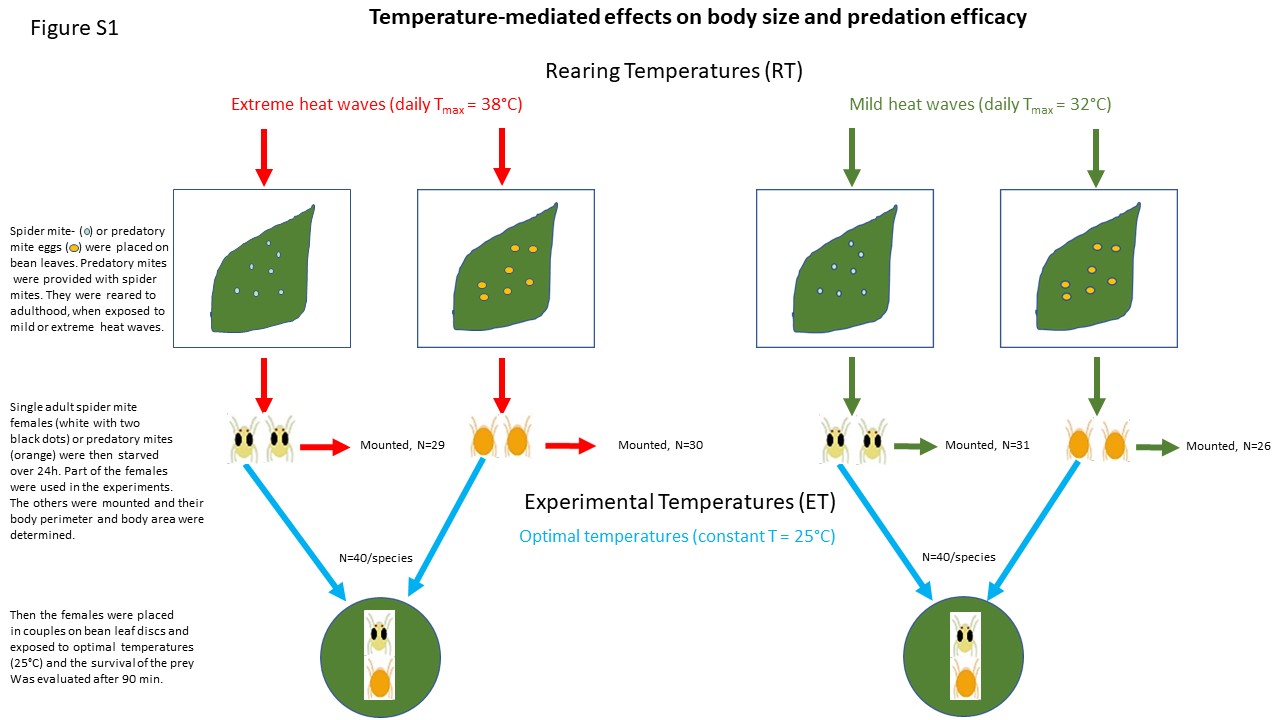

Supplement: Supplementary file 1 — Figure S1: Experimental set‐up of experiment 1: Temperature‐mediated effects on body size and predation efficacy. Figure S2: Experimental set‐up of experiment 2: Predation under heat stress. Data S1: ece373156‐sup‐0001‐supinfo.zip. [file ECE3-16-e73156-s001.zip › ece373156-sup-0001-FigureS1.jpg]

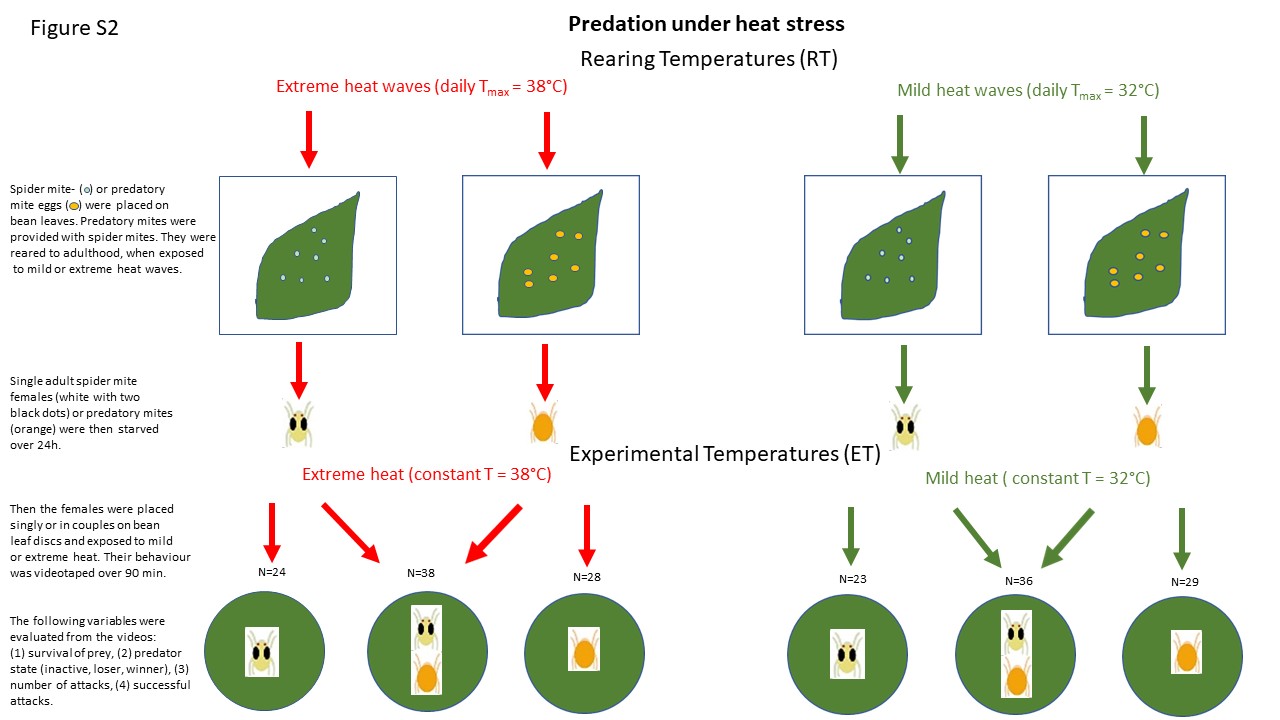

Supplement: Supplementary file 1 — Figure S1: Experimental set‐up of experiment 1: Temperature‐mediated effects on body size and predation efficacy. Figure S2: Experimental set‐up of experiment 2: Predation under heat stress. Data S1: ece373156‐sup‐0001‐supinfo.zip. [file ECE3-16-e73156-s001.zip › ece373156-sup-0002-Figure_S2.jpg]
